# Single-Cell Genome and Group-Specific *dsrAB* Sequencing Implicate Marine Members of the Class *Dehalococcoidia* (Phylum *Chloroflexi*) in Sulfur Cycling

**DOI:** 10.1128/mBio.00266-16

**Published:** 2016-05-03

**Authors:** Kenneth Wasmund, Myriel Cooper, Lars Schreiber, Karen G. Lloyd, Brett J. Baker, Dorthe G. Petersen, Bo Barker Jørgensen, Ramunas Stepanauskas, Richard Reinhardt, Andreas Schramm, Alexander Loy, Lorenz Adrian

**Affiliations:** a Helmholtz Centre for Environmental Research—UFZ, Leipzig, Germany; b Division of Microbial Ecology, Department of Microbiology and Ecosystem Science, Research Network Chemistry meets Microbiology, University of Vienna, Vienna, Austria; c Department of Bioscience, Center for Geomicrobiology, Aarhus University, Aarhus, Denmark; d Department of Marine Science, University of Texas-Austin, Marine Science Institute, Port Aransas, Texas, USA; e Bigelow Laboratory for Ocean Sciences, East Boothbay, Maine, USA; f Max Planck Genome Centre Cologne, Cologne, Germany

## Abstract

The marine subsurface sediment biosphere is widely inhabited by bacteria affiliated with the class *Dehalococcoidia* (DEH), phylum *Chloroflexi*, and yet little is known regarding their metabolisms. In this report, genomic content from a single DEH cell (DEH-C11) with a 16S rRNA gene that was affiliated with a diverse cluster of 16S rRNA gene sequences prevalent in marine sediments was obtained from sediments of Aarhus Bay, Denmark. The distinctive gene content of this cell suggests metabolic characteristics that differ from those of known DEH and *Chloroflexi*. The presence of genes encoding dissimilatory sulfite reductase (Dsr) suggests that DEH could respire oxidized sulfur compounds, although *Chloroflexi* have never been implicated in this mode of sulfur cycling. Using long-range PCR assays targeting DEH *dsr* loci, *dsrAB* genes were amplified and sequenced from various marine sediments. Many of the amplified *dsrAB* sequences were affiliated with the DEH Dsr clade, which we propose equates to a family-level clade. This provides supporting evidence for the potential for sulfite reduction by diverse DEH species. DEH-C11 also harbored genes encoding reductases for arsenate, dimethyl sulfoxide, and halogenated organics. The reductive dehalogenase homolog (RdhA) forms a monophyletic clade along with RdhA sequences from various DEH-derived contigs retrieved from available metagenomes. Multiple facts indicate that this RdhA may not be a terminal reductase. The presence of other genes indicated that nutrients and energy may be derived from the oxidation of substituted homocyclic and heterocyclic aromatic compounds. Together, these results suggest that marine DEH play a previously unrecognized role in sulfur cycling and reveal the potential for expanded catabolic and respiratory functions among subsurface DEH.

## IMPORTANCE

Sediments underlying our oceans are inhabited by microorganisms in cell numbers similar to those estimated to inhabit the oceans. Microorganisms in sediments consist of various diverse and uncharacterized groups that contribute substantially to global biogeochemical cycles. Since most subsurface microorganisms continue to evade cultivation, possibly due to very slow growth, we obtained and analyzed genomic information from a representative of one of the most widespread and abundant, yet uncharacterized bacterial groups of the marine subsurface. We describe several key features that may contribute to their widespread distribution, such as respiratory flexibility and the potential to use oxidized sulfur compounds, which are abundant in marine environments, as electron acceptors. Together, these data provide important information that can be used to assist in designing enrichment strategies or other postgenomic studies, while also improving our understanding of the diversity and distribution of *dsrAB* genes, which are widely used functional marker genes for sulfur-cycling microbes.

## INTRODUCTION

Microorganisms are the primary drivers of elemental cycles within marine subsurface sediments, playing key roles in the mineralization of organic matter derived from the overlaying water column and the recycling of nutrients to the water column. Microorganisms thus control the extent of burial of organic matter into the deep subsurface and thereby influence the long-term sequestration of carbon from the oceans. The microbial communities that mediate these processes are therefore fundamental catalysts of global element and energy cycles (1–3). Despite the far-reaching significance of microbes inhabiting marine sediments, little is known about the physiologies of the many major taxonomic groups inhabiting these environments. Culture-independent molecular surveys of microbial life in marine sediments have continuously demonstrated the presence of members of the phylum *Chloroflexi*, which are widely distributed (4, 5) and yet poorly understood with regard to their basic modes of living, i.e., their sources of nutrients and energy or mechanisms to conserve energy.

The *Dehalococcoidia* (DEH), a class-level phylogenetic clade within the phylum *Chloroflexi* (6), are a particularly widespread group in the marine subsurface (7–16). Members of the DEH are known to exist at locations from shallow sediments few centimeters below the surface to deep subsurface sediments hundreds of meters below the seafloor; they often dominate communities in deep energy-limited sediments (4, 5). Cultivated members of the DEH, i.e., strains of the genera *Dehalococcoides*, *Dehalogenimonas*, and *Candidatus* “Dehalobium,” phylogenetically cluster in a distinct clade within the class DEH. All cultured members of the DEH are highly specialized organisms that grow via organohalide respiration by using hydrogen or formate as an electron donor and halogenated organics as terminal electron acceptors (6). Importantly, however, phylogenetic analyses of DEH inhabiting marine sediments consistently show the coexistence of remarkably diverse DEH that are divergent from cultured DEH species (12, 16, 17). Phylotypes affiliated with cultured DEH are present in low relative abundance compared to other DEH, which indicates that phylotypes that can be more confidently linked to organohalide respiration are not abundant members of microbial communities in marine sediments (16). The relative abundances of the various DEH subgroups change with depth and differ between sites (16). This suggests that different biogeochemical conditions strongly influence distributions of different subgroups and implies that varied metabolic properties exist among the different subgroups.

The hypothesis that various metabolic potentials exist among the DEH was recently supported by analyses of single-cell-derived genomes (DEH-J10, Dsc1, and DscP2) (18, 19) and metagenome-derived genomes (RBG-2 and RBG-1351) (20). These studies revealed highly divergent genome structures and metabolic potential in comparison to cultivated DEH. Key metabolic properties predicted from those genomic analyses include the potential for oxidizing fatty acids and the ability to produce ATP via the formation of acetate, properties that could not have been predicted based on phylogenetic relationships with cultured members of the DEH. Furthermore, no evidence for genes related to organohalide respiration was identified in the genomic data, suggesting that DEH that are not closely related to isolated strains use other mechanisms to conserve energy. Although the aforementioned genome-based studies have elucidated the metabolic potential of several uncultured DEH, it is clear that extensive evolutionary diversity exists within the DEH and that the previous studies had merely begun to scratch the surface regarding the genomic diversity and metabolic potential of DEH. It is therefore imperative to study additional genomes of DEH derived from the various undescribed clades to better understand their metabolic diversity and different biogeochemical roles and the different ecological niches that may be occupied by the various members of this class.

In this report, we provide a detailed description of the genomic content of a single DEH cell (DEH-C11) originating from marine sediments. The bacterium was divergent from cultured DEH species and previously analyzed DEH single-cell genomes and exhibited various features not previously described in the class DEH or the phylum *Chloroflexi*. Among these, evidence for multiple respiratory mechanisms distinguishes this organism from other studied DEH. Furthermore, the detection of reductive, bacterial-type *dsrAB*, which is suggestive of sulfite reduction and possibly sulfate reduction (although genes for sulfate activation or reduction to sulfite were not identified), provides evidence of participation of the marine DEH in the sulfur cycle. On the basis of these genomic results, we further investigated the presence of DEH-derived *dsrAB* in marine sediments by PCR after developing and applying primers for DEH-related *dsr* amplification, cloning and sequencing. This demonstrated that diverse DEH-related *dsrAB* genes are present in various marine sediments. We also provide important information detailing possible nutrient sources for the organism which can aid future cultivation strategies.

## RESULTS AND DISCUSSION

### Phylogeny and distribution of single-cell DEH-C11 in Aarhus Bay sediments.

**FIG 1:**
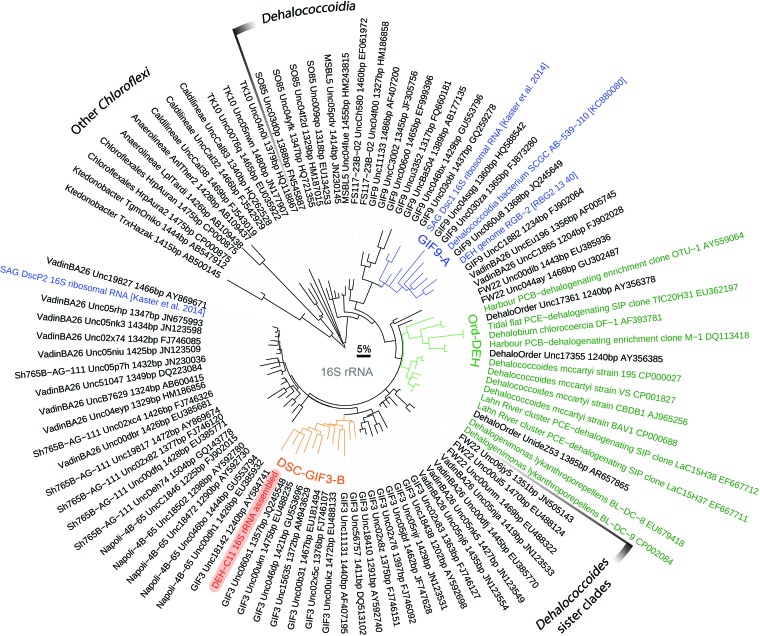
Phylogenetic analysis of the 16S rRNA gene from DEH-C11 in comparison to those of other members of the class DEH. The tree is based on the maximum likelihood algorithm. Branches in orange highlight the DSC-GIF3-B subgroup, to which the 16S rRNA gene of DEH-C11 (leaf label highlighted in red) is affiliated. Branches in green highlight the clade containing all known organohalide-respiring phylotypes, and leaf labels of sequences from cultivated bacteria or sequences implicated in organohalide respiration are highlighted in green. Branches in blue highlight the GIF9-A clade. Leaf labels in blue highlight sequences for which genomic information is available (18–20). The scale bar represents 5% sequence divergence. PCB, polychlorinated biphenyl; PCE, tetrachloroethene; SIP, stable isotope probing.

Single cells from marine sediments (10 cm below sea floor [cmbsf]) of Aarhus Bay, Denmark, were isolated by flow cytometry. After multiple-displacement amplification (MDA) and 16S rRNA gene sequencing, DNA from a single bacterial cell (designated “DEH-C11”) that was divergent from cultured members of the DEH, single amplified genomes (SAGs), and metagenomic bins was selected for deep sequencing and analyses of gene content. The cell affiliated with the previously defined “DSC-GIF3-B” subgroup of the DEH (Fig. 1) (16), which also falls within the “*Dehalococcoides* sister clades” (17). Sequences belonging to these *Dehalococcoides* sister clades are among the most prevalent DEH sequences detected in marine sediments (16). The 16S rRNA sequence of DEH-C11 is 88% similar to that of *Dehalogenimonas lykanthroporepellens* strain BL-DC-9, the most closely related cultivated strain, which approximately equates to family-level divergence (21). It is also highly divergent from previously described single-cell genomes (18, 19) or metagenome-derived genomes (20), which affiliate with completely different clusters of the DEH, based on 16S rRNA phylogeny (Fig. 1). Although the metagenomic bin of RBG-1351 does not contain a 16S rRNA gene (20), our comparisons of bidirectional BLASTP hits of all predicted proteins revealed that only 49% of predicted proteins from DEH-C11 had homologs in the genome of RBG-1351 and that, of these, an average protein sequence identity of only 55% was determined. This demonstrates that the degree to which the DEH-C11 genome is distinct from those of other DEH is considerable, and this is reflected in its metabolic potential compared to that of other DEH (described below). DEH subgroup DSC-GIF3 accounted for 4% of the 16S rRNA genes recovered by PCR amplification with DEH-targeted primers from all depths (10 to 300 cmbsf) of the Aarhus Bay site from which the cell had been isolated (16).

### Genomic data obtained from single-cell DEH-C11.

All reads from sequencing of MDA-derived DNA were assembled into 233 contigs of greater than 1,000 bp. After removal of contigs that did not contain genes with highest similarity to genes of *Chloroflexi*, a “high-confidence” assembly of 0.94 Mbp within 155 contigs was obtained with an average length of 6.1 kbp (Table 1). Pentanucleotide analysis of the nucleotide coding signatures of contigs showed that all but three contigs from the high-confidence DEH-C11 data set clustered together (see Fig. S1 in the supplemental material), further corroborating that the data set originated from a single genome. The high-confidence data set represented approximately 42% to 46.8% of the estimated full genome of 2.01 to 2.24 Mbp (Table 1). This is considerably larger than the streamlined genomes of the metabolically specialized, cultivated genera *Dehalococcoides* and *Dehalogenimonas*, which range from 1.34 to 1.69 Mbp (22, 23). This gave a first hint that the DEH-C11 organism may encode expanded metabolic capabilities compared to its closest cultivated relatives.

**TABLE 1:** Assembly statistics of the DEH-C11 genome content

Parameter	High-confidence data set value(s)
Assembly size (Mbp)	0.94
Avg GC content (%)	47.9
No. of contigs	155
Mean contig length (kbp)	6.1
Longest contig length (kbp)	35.4
No. of predicted CDS	990
Estimation of % genome recovered	
tRNA	42
CSCG	42–43
CheckM	46.8
Genome size estimation (Mbp)	2.01–2.24

### Sulfite reduction as a potential mode of energy conservation in *Chloroflexi*. (i) Dissimilatory sulfite reductase in the genome of DEH-C11.

**FIG 2:**
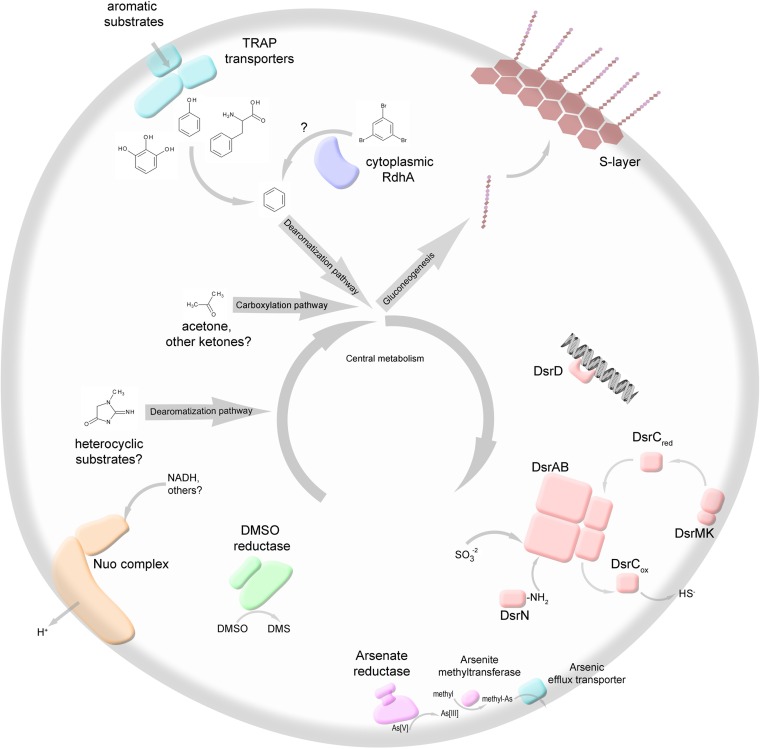
A simplified schematic of putative biochemical properties predicted from genomic content of DEH-C11. The redox cycling schematic for sulfite reduction is based on model proposed by Venceslau et al. (24). Aromatic substrates depicted are (left to right) phenylalanine, phenol, and pyrogallol. The heterocyclic compound depicted is creatinine. Nuo, NADH:ubiquinone oxidoreductase complex; TRAP, tripartite ATP-independent periplasmic transporters; DMSO, dimethyl sulfoxide; DMS, dimethyl sulfide; Dsr, dissimilatory sulfite reductase; S-layer, surface-layer protein coat.

**FIG 3:**
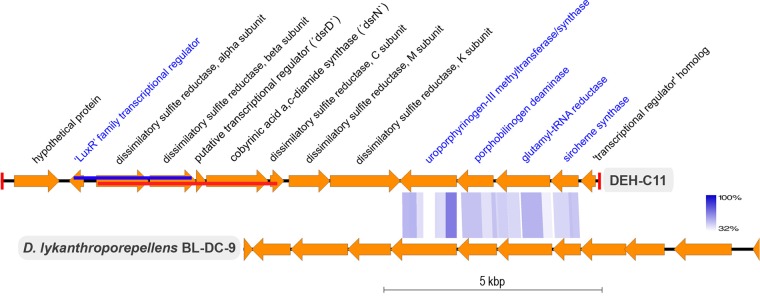
Representation of the gene order present on the contig (IDBA scaffold 11) containing the dissimilatory sulfite reductase operon and associated genes from DEH-C11 (top) and *D. lykanthroporepellens* BL-DC-9 (bottom). Gene names in blue denote that the best BLASTP hits were to *D. lykanthroporepellens* BL-DC-9 or other *Chloroflexi* strains. Blue shaded lines between the two gene order representations show regions with high sequence similarity and synteny determined by tBLASTx as implemented using EasyFig (93). Degrees of tBLASTx sequence identity are depicted in the colored legend. The red blocks at the end of the DEH-C11 contig representation denote the ends of the contig sequence. The red and blue lines within the genes of the DEH-C11 represent the regions of the genome amplified by long-range PCR.

A cluster of genes encoding a dissimilatory sulfite reductase (Dsr) complex and accessory subunits was identified in an assembled part of the DEH-C11 genome (Fig. 2 and 3). Two lines of evidence indicate that this gene cluster genuinely originated from the DEH-C11 genome. First, genes most closely related to *Dehalogenimonas lykanthroporepellens* BL-DC-9 genes flank the *dsr* cluster (Fig. 3). Second, pentanucleotide signature analysis shows that the *dsr*-harboring contig clusters closely with all other DEH-C11 contigs (see Fig. S1 in the supplemental material). The cluster contains the genes *dsrABCMK*, which encode a full complement of the Dsr subunits required for the reduction of sulfite (24) (Fig. 2 and 3; see also Table S1 in the supplemental material). Two additional genes, *dsrD* and *dsrN*, commonly present in *dsr* operons and encoding proteins not directly involved in electron shuttling during sulfite reduction, were also present in the cluster. The genes *dsrD* and *dsrN* encode a putative transcriptional regulator and an enzyme involved in the amidation of the sirohemes of DsrAB, respectively. No genes encoding DsrJOP proteins, which are typically found in Gram-negative but not in Gram-positive sulfate reducers, were present. Instead, Gram-positive bacteria are thought to use DsrMK only to transfer electrons from the menaquinone pool to DsrC (24). The presence of conserved cysteine residues required for catalytic sites in the encoded DsrAB and DsrC subunits suggests that the encoded enzymes were functional.

A dissimilatory direction was predicted by the presence of a gene for DsrD, which appears to be specific for sulfite-reducing organisms, since it is absent in all known sulfur-oxidizing organisms that use Dsr in the reverse direction (25). A reductive direction is also supported by the phylogenetic placement of the DsrAB in the reductive branch of the DsrAB phylogenetic tree (described further below). The presence of a *dsr* cluster with all genes required for a functional complex, as well as conserved amino acid residues in the key enzymes indicative of active enzymes, provided evidence that DEH-C11 could have used sulfite for anaerobic respiration. Sulfite could be utilized directly, since it is a common “intermediate” of the sulfur cycle (26), or could be enzymatically derived from sulfate, thiosulfate, or sulfonated organics. Genes for enzymatic transformations from sulfate (i.e., activation via adenosine 5′-phosphosulfate) were not identified in the assembly or in unassembled reads, and no genes indicating that sulfite could be derived from thiosulfate or sulfonated organics were identified in the assembled genome. Additional sequencing of related genomes will be required in order to resolve the issue regarding the source of sulfite for these organisms. Although *dsrAB* genes have been identified in syntrophic *Desulfotomaculum* strain MGP (27) and thiosulfate-disproportionating *Desulfocapsa sulfexigens* strain SB164P1 (*Desulfobulbaceae*) (28), which appear to have recently lost the ability to respire sulfate, these strains are rare among their relatives. That is, most known members of the genus *Desulfotomaculum* and the family *Desulfobulbaceae* have the ability to reduce sulfate. It is therefore plausible that even if DEH-C11 were not capable of sulfite reduction due to a recent change in lifestyle, other relatives of the organism might still be capable of sulfite reduction.

Detection of *dsr* genes in DEH bacteria indicates a potential for sulfite reduction in members of the phylum *Chloroflexi* and suggests a previously unexpected role for these bacteria in marine sulfur cycling. It may also provide hints with regard to the widespread nature of DEH within marine environments, since they are rich in sulfur compounds. A recent study reported the presence of genes for DsrAB in a metagenome-derived genome of an *Anaerolineae* (*Chloroflexi*) organism and suggested these organisms might therefore be capable of sulfate reduction (29). Our examination of the encoded proteins for comparative purposes identified these as homologs of small iron-sulfur-containing ferredoxin-like proteins, most similar to proteins from methanogens, while no genes for bona fide DsrAB and DsrC (key catalytic enzymes required for sulfite reduction) were found. We therefore conclude that those *Anaerolineae*-affiliated organisms may not have the genetic potential for dissimilatory sulfite or sulfate reduction.

Because the reduction of oxidized sulfur compounds drives the oxidation of vast amounts of organic matter in marine sediments on a global scale (30, 31), a contribution to this process by widespread DEH could be biogeochemically significant. The abundance of DEH-related *dsrAB* relative to total *dsrAB* in marine sediments could not be estimated reliably, since previously used *dsrAB* primers contained up to 6 mismatches to the *dsrAB* of DEH-C11 (32, 33). This would therefore lead to severe underestimation of the relative abundances of DEH-related *dsrAB* genes. Future studies using updated primer sets targeting *dsrAB* or metagenomic data are required to gain reliable estimates of the relative abundances of DEH-related *dsrAB* genes and of their potential contribution to sulfur cycling in marine sediments.

**FIG 4:**
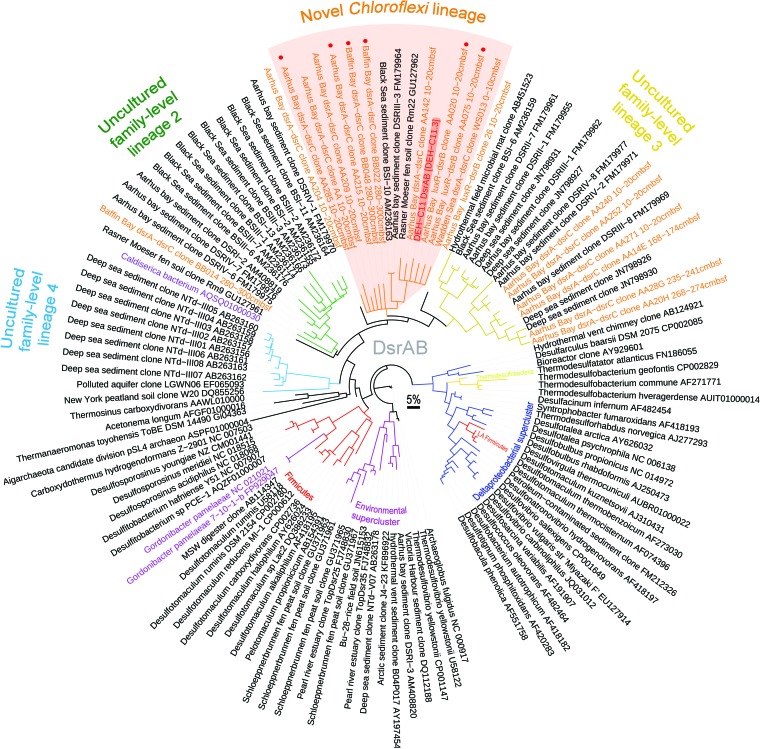
Phylogenetic analysis of DsrAB from DEH-C11 (highlighted in red) and cloned DsrAB sequences as determined by the evolutionary placement algorithm (88), which was used to place sequences onto a previously constructed DsrAB consensus tree (34). Orange leaf labels indicate sequences retrieved by long-range PCR in this study. L.A.-*Firmicutes*, DsrAB proteins that were laterally acquired by certain *Firmicutes* species of the *Deltaproteobacteria* (34). The red dots on leaf labels indicate that the DsrAB sequences are derived from amplified sequences that were sequenced by primer walking and with pentanucleotide signatures similar to that of the DEH-C11 genome (see Fig. S1 in the supplemental material). The grey scale bar represents 5% sequence divergence.

Phylogenetic analysis of the DsrAB sequence of DEH-C11 showed that it is a member of a sister clade to the recently denominated “uncultured family-level lineage 3” (Fig. 4) (34). The DEH-C11-related clade, along with uncultured family-level lineages 2, 3 and 4, is composed almost entirely of DsrAB sequences retrieved by PCR-based approaches from unidentified microorganisms inhabiting marine sediments (32, 33, 35, 36). The aforementioned clades are also part of the broadly defined “*Firmicutes*-related” sensu lato group (34).

The *dsr* operon in DEH-C11 is flanked by genes encoding proteins involved in the synthesis of a siroheme cofactor required by DSR for catalytic activity, i.e., genes stretching from the uroporphyrinogen-III methyltransferase/synthase gene to the siroheme synthase gene (Fig. 3) (37). Intriguingly, predicted protein sequences for synthesis of the siroheme cofactor are most similar to homologs from *Dehalogenimonas lykanthroporepellens* BL-DC-9, a cultivated member of DEH (Fig. 3). This finding therefore opens an evolutionary question as to whether *Dehalogenimonas* and other related DEH previously harbored the genetic capacity for sulfite reduction. The lack of other genes required for sulfite reduction in *Dehalogenimonas lykanthroporepellens* BL-DC-9 might be the result of genomic streamlining due to a more contemporary specialization for organohalide respiration.

### (ii) Amplification of putative DEH-related *dsrAB* from marine sediments.

To investigate if *dsr* genes can be found in other DEH-related bacteria in marine sediments and to explore further the diversity of *dsr* genes in DEH, we designed degenerate primer sets to directly amplify DEH-related *dsr* loci from DNA extracted from different sediment sites. The aim was to amplify “long” (up to 3.9 kbp) DNA sequences containing novel DEH-C11-related *dsrAB* sequences and flanking DEH-related “marker” genes in order to support the phylogenetic affiliation of the amplified *dsrAB* sequences. As flanking marker genes, a gene for a siroheme cofactor synthesis enzyme and a gene for a LuxR-related “transcriptional regulator,” both with highest sequence similarity to genes in *Dehalogenimonas* spp. (Fig. 3), were targeted. With primers targeting *luxR* and *dsrB*, a 2.5-kbp fragment (Fig. 3) was successfully amplified from sediments of Aarhus Bay. The retrieved clone sequences were closely related to *dsrAB* of DEH-C11, showing nucleotide sequence identities of 97% to 100% (Fig. 4; see also Table S2 in the supplemental material). Although no products were obtained in attempts to amplify fragments with genes for siroheme cofactor synthesis enzymes and *dsrAB*, two alternative degenerate primer pairs targeting *dsrA* and *dsrC* that amplify *dsrABDNC* (Fig. 3) proved to successfully amplify ~3.9-kbp fragments and enabled recovery of the more divergent DEH-C11-related *dsrAB* sequences (Fig. 4). Sixteen end-sequenced clones obtained from different sediment depths of Aarhus Bay at the location where DEH-C11 was isolated, as well as from the Baffin Bay, Greenland and from tidal flat sediments of the Wadden Sea, Germany (Fig. 4), featured *dsrA* nucleotide sequences that were unique; i.e., they were not found in *dsrA* of DEH-C11. Only two sediment samples that were tested failed to provide amplicons, namely, sediments from the Peru margin and from the Black Sea. Phylogenetic placement of DsrA or DsrB sequences onto a previously constructed consensus reference tree (34) by the evolutionary placement algorithm (EPA), as well as by *de novo* constructed trees based on partial DsrA sequences using both neighbor-joining and maximum likelihood algorithms (results not shown), revealed that eight sequences formed a monophyletic clade with the DsrAB of DEH-C11 (Fig. 4). Based on the distinct monophyletic clustering of the eight clone sequences with DsrA of DEH-C11 compared to other stable family-level lineages as shown by using all three approaches and on the fact that the best BLASTX hits of corresponding *dsrN* sequences were to genes encoding the DsrN of DEH-C11 (see Table S1), we infer that these clones are likely derived from DEH organisms. Six clones from the monophyletic DEH-C11-related clade were also further sequenced by primer walking to obtain sufficient sequence information for nucleotide signature analysis. These clones showed pentanucleotide signatures very similar to those of DEH-C11 (see Fig. S1), suggesting DEH-related bacteria as their sources.

Some of the obtained *dsrAB* sequences of the monophyletic DEH-C11-related clade shared as little as 76% nucleotide sequence identity with the *dsrAB* of DEH-C11, while minimum intragroup sequence identities of 73% for nucleotide sequences and 86% for amino acid sequences were determined (see Table S1 in the supplemental material). The minimum intragroup sequence identity determined here is well within the range of intrafamily amino acid sequence identities of known families of sulfate-reducing microorganisms, which range from 64% to 89% (34, 38). On the basis of previously constructed linear regression plots of corresponding pairs of 16S rRNA and *dsrAB* genes derived from many sequenced isolates (34), we deduced that the organisms harboring the cloned DEH-related *dsrAB* genes with 76% nucleotide sequence identity to DEH-C11 may harbor 16S rRNA genes with less than 94.5% identity (see Fig. S2). This is below a recently defined genus-level taxonomic threshold (21). It could therefore be inferred that the organisms harboring these genomic fragments belong to different genera, and we propose that a family-level clade within the DEH exists that harbors genes for Dsr. No evidence for DEH-related *dsr* could be identified in the metagenomes searched. Ongoing research aims to uncover the diversity of *dsr* in DEH and to determine whether other genes can be identified that may indicate the source of intracellular sulfite.

### Other respiratory modes inferred from genome annotations. (i) Molybdenum-containing oxidoreductases.

Four genes encoding catalytic alpha subunits of complex iron-sulfur molybdoenzyme (CISM) oxidoreductases (39) that may function as terminal reductases were present in the genome of DEH-C11. Phylogenetic analysis showed that two CISM alpha subunits from DEH-C11 affiliated with known dimethyl sulfoxide (DMSO)-reducing enzymes and branched closest to homologs from another DEH single-cell genome, that of DEH-J10 (see Fig. S3 in the supplemental material) (19). DMSO and trimethylamine *N*-oxide (TMAO) were previously discussed as candidate substrates for these predicted enzymes (19), although experimental validation is needed. One of the predicted CISM alpha subunits (DEH-C11.384) had no close phylogenetic affiliation, while another (DEH-C11.399) was affiliated with the broad arsenate/polysulfide/thiosulfate oxidoreductase branch of the CISM phylogeny. A putative role as an arsenate reductase alpha subunit was inferred by various lines of evidence, including the presence of a twin-arginine transport (TAT) peptide “leader” signal sequence predicted to guide the subunit to or across the cytoplasmic membrane, which thereby distinguishes it from the cytoplasmic arsenate reductases typically used for resistance to arsenic toxicity (40, 41). Additionally, colocalization of genes encoding an arsenical resistance operon repressor (ArsR family), as well as those encoding an adenosylmethionine-dependent methyltransferase and an arsenite efflux transporter, which may act to methylate and extrude the reduced and toxic As(III) from the cell, strongly suggests that this enzyme could reduce arsenate (42) (Fig. 2). Further genes encoding homologs of formate dehydrogenase-like (FDH) beta and gamma subunits were present in adjacent locations and may function to transfer electrons and embed the complex into the membrane, respectively, if analogous to other known three-subunit CISM complexes (43) (Fig. 2). Although arsenic resistance genes were previously identified in other DEH genome data, i.e., the RBG-2 and RBG-1351 metagenome-derived genomes (20), this is to our knowledge the first report of a potential respiratory arsenate reductase in DEH and also in the phylum *Chloroflexi*.

Although very little is known about the existence and activity of microorganisms involved in arsenic cycling in marine sediments, various arsenic species are naturally prevalent in marine waters and sediments, even in marine environments that are free from direct anthropogenic impact (44). Recent metagenomic analyses have identified genes encoding enzymes predicted to catalyze transformations in the arsenic cycle, including arsenate reduction, in various marine sediments (45, 46). Our results therefore indicate that so-far-overlooked arsenic cycling microorganisms occur in marine sediments and suggest that microorganisms capable of performing such transformations fill a functional niche in marine sediments.

### (ii) Reductive dehalogenase homologous genes.

A single gene encoding a reductive dehalogenase subunit A homolog (RdhA) was identified in the DEH-C11 genomic content. The predicted enzyme contains conserved cysteine residues required for two Fe-S cluster binding motifs typical of known RdhA enzymes, suggesting that the encoded enzyme was functional. No TAT peptide leader signal sequence required for protein translocation across the cytoplasmic membrane was detected. Furthermore, no genes were identified for predicted membrane-anchoring RdhB subunits, which are often encoded by sequences adjacent to *rdhA*. Other *rdhA* genes that do not encode TAT sequences and without adjacently encoded RdhB have been previously found, e.g., in *D. lykanthroporepellens* BL-DC-9 (23) and in the aerotolerant nonrespiratory reductive dehalogenase of *Nitratireductor pacificus* pht-3B (NprdhA) (47).

**FIG 5:**
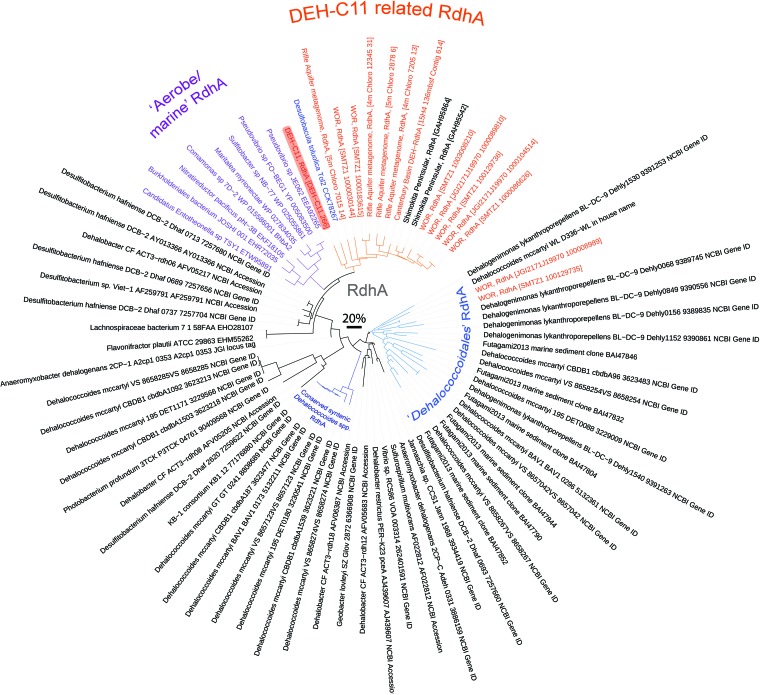
Phylogenetic analysis of RdhA derived from DEH-C11 (highlighted in red), DEH-derived RdhA retrieved from metagenomes, and RdhA from pure reference strains. The tree is based on the maximum-likelihood algorithm. Branches of metagenome-derived RdhA sequences determined to originate from DEH- or *Chloroflexi*-related contigs are highlighted in orange (50, 51, 74). Branches corresponding to the “conserved syntenic” *Dehalococcoides* species RdhA are highlighted in dark blue, while the *Dehalococcoidales* RdhA clade are highlighted in light blue. Nearly full-length RdhA sequences from the Smkt-3 clade (two of six available were nearly full length) from subsurface sediments of Shimokita peninsular were included (49). Branches of RdhA derived from aerobic bacteria are highlighted in purple. The grey scale bar represents 20% sequence divergence. WOR, White Oak River.

The DEH-C11 RdhA sequence has low sequence similarity to known RdhA enzymes used for respiration by cultivated DEH organisms. Instead, it is most similar to a homolog derived from *Desulfobacula toluolica* Tol2, an aromatic-compound-degrading sulfate reducer isolated from marine sediments, which to our knowledge has not been tested for growth via organohalide respiration (48). The RdhA sequence in strain Tol2 also does not contain a TAT leader peptide and has no adjacently encoded RdhB. Phylogenetic analysis shows the DEH-C11 RdhA forms a clade with (i) the RdhA of *D. toluolica* Tol2, (ii) clade “Smkt-3” RdhA from metagenomic contigs of unknown phylogenetic origin derived from marine sediments of Shimokita Peninsula, Japan (49); (iii) RdhA sequences from *Chloroflexi*-binned metagenomic scaffolds from a terrestrial aquifer sediment-derived metagenome (20); (iv) a single RdhA from a contig (that we deduced is derived from a DEH organism) from a metagenome of deep subsurface sediments of the Canterbury Basin, New Zealand (50); and (v) RdhA sequences from estuary sediment-derived metagenomic contigs (51), which we identified as belonging to DEH or *Chloroflexi* in this study (Fig. 5). The clade of mostly DEH-derived RdhA is distinct from previously described clades of RdhA derived from DEH such as the “conserved sytenic” RdhA of *Dehalococcoides* spp., as well as the diverse “*Dehalococcoidales*” RdhA clade (52) (Fig. 5). In 8 of the 12 *rdhA*-containing metagenomic contigs analyzed, we observed the gene for tRNA-His-GTG (and often also the gene for tRNA-Arg-TCG) adjacent to *rdhA* and yet observed few other syntenic genes, hinting at only a small degree of conservation among the genomes (see Fig. S4 in the supplemental material). Only two RdhA sequences from the estuary sediment-derived metagenomic contigs (51), which were derived from nearly identical contigs, affiliated with the *Dehalococcoidales* RdhA clade (Fig. 5). The DEH-C11-related RdhA clade is a sister clade to RdhA derived from various aerobic marine bacteria and includes strains such as *Comamonas* sp. strain 7D-2 and *N. pacificus* pht-3B, which have been described to contain nonrespiratory reductive dehalogenases (47, 53). Additionally, a MarR-type transcriptional regulator was encoded by a gene directly adjacent to *rdhA* in DEH-C11, and the gene was most similar in sequence to MarR regulator genes from cultivated DEH that are typically associated with regulation of *rdhA* expression (22).

Together, the results indicate that the RdhA in DEH-C11 was located in the cytoplasm and was therefore not likely involved in a membrane-bound respiratory chain. Possible alternative functions include the reoxidation of respiratory cofactors for a “facilitated fermentation,” detoxification of substrates, or the removal of halogens from organics to enable further catabolism. Dehalogenation of substrates to enable further catabolism was recently described for the aerobic strains *Comamonas* sp. strain 7D-2 and *N. pacificus* pht-3B, which dehalogenate aromatic compounds prior to further catabolic processing (47, 53). The phylogenetic relatedness of the RdhA of DEH-C11 to the RdhA of *Comamonas* sp. strain 7D-2 and *N. pacificus* pht-3B could suggest similar functions for these enzymes in anaerobes, although such functions will need to be experimentally verified. The apparent high frequency of *rdhA* (49) and of haloacid dehalogenase genes (50) in the genetic content of marine subsurface microbes suggests that there is an evolutionary advantage to possessing the capacity to dehalogenate organics in marine subsurface environments.

### (iii) NADH-ubiquinone oxidoreductase.

The DEH-C11 bacterium encoded an 11-subunit NADH-ubiquinone oxidoreductase (nuo), while it lacked genes for a cytoplasmic electron input “N-module," i.e., NuoEFG subunits. This is analogous to genomes of cultured DEH that encode similar 11-subunit Nuo complexes but that were hypothesized to function in combination with an archaeon-like cofactor F420 input module (FpoF) (54). The predicted complex may play a role in anaerobic respiration by transferring reducing equivalents from cytosolic oxidation reactions to an electron transport chain that is connected with proton translocation. The Nuo complex was incomplete in the genome recovery of the metagenome-derived genome of RBG-1351, which gave the first described genomic information derived from the *Dehalococcoides* sister clades (20). In combination with a lack of genes for terminal respiratory reductases in RBG-1351, this led the authors to infer that the organism was likely an obligate fermentative organism and that fermentative metabolism may be widespread among the DEH, since genomic content from other DEH clades (i.e., GIF-9) also suggested fermentative lifestyles (19, 20). In a further search, we identified several other full *nuo* gene clusters from the previously generated Rifle Aquifer metagenome (20) that were most similar in protein sequence identity to those of cultured DEH, single-cell DEH-C11, and metagenome-derived genome RBG-1351 (results not shown). This similarity was exemplified by phylogenetic analysis of corresponding NuoL subunits (the largest subunit of the Nuo complex) from the full *nuo* gene clusters from the metagenome and reference *Chloroflexi* (see Fig. S5 in the supplemental material). Together, these results provide evidence that members of the *Dehalococcoides* sister clades are not limited to fermentative modes of energy conservation.

### Electron donors. (i) Catabolism of homocyclic aromatics.

Multiple genes provided evidence that the DEH-C11 bacterium had the capacity to oxidize aromatic organic molecules with various substituents. These include three copies of genes encoding pyrogallol hydroxytransferases similar in amino acid sequence to predicted homologs from bacteria known to degrade hydroxylated aromatic molecules (see Fig. S3 in the supplemental material) (55, 56). Pyrogallol hydroxytransferases could enable the bacterium to perform transhydroxylation or hydroxylation reactions required for catabolism of aromatic molecules with metapositioned hydroxyl groups (57). Genes encoding pyrogallol hydroxytransferases were also previously identified in the metagenome-derived genomes of RBG-1351 and RBG-2 (20).

Genes required for catabolism of aromatics without metapositioned hydroxyl groups were also identified. These included genes encoding two subunits of a putative benzylsuccinate coenzyme A (CoA) transferase (DEH-C11.996 and DEH-C11.997), which have also been identified in DEH-J10 and RBG-1351. This indicates the DEH-C11 cell could activate aromatics to arylcarboxyl-CoA esters, which could be further catabolized via a central ring reduction and cleavage pathway. Downstream were two genes encoding alpha and delta subunits (DEH-C11.982 and DEH-C11.983) of a phenylphosphate carboxylase-like enzyme, which is one of the key enzymes for activation of phenol to benzoyl-CoA. Further genes identified that may be related to enzymes catalyzing steps of phenol degradation include candidate genes for a putative 4-hydroxybenzoyl-CoA reductase (A, B, and C subunits), although this enzyme is also known to function in analogous steps in degradation pathways of other substituted aromatic molecules (58). Genes encoding subunits of a phenylglyoxylate:acceptor oxidoreductase (DEH-C11.203-205) and a phenylacetyl-CoA:acceptor oxidoreductase (not present in the high-confidence data set) indicate that an anaerobic phenylacetate degradation pathway exists which could facilitate the degradation of the aromatic amino acid phenylalanine. Degradation of phenylalanine appears to be common among aromatic compound-degrading anaerobes (58), since the carbon can be funneled into the benzoyl-CoA degradation pathway used for the final breakdown of aromatic molecules.

An additional indication of the uptake and catabolism of aromatic molecules was found after examination of BLASTP results that revealed numerous gene clusters for tripartite ATP-independent periplasmic (TRAP) transporters with highest amino acid sequence identities to encoded proteins from well-characterized aromatic compound-degrading bacteria. For instance, five of eight TRAP “solute-binding” P subunits were most similar to protein sequences from the polyaromatic-degrading deltaproteobacterial strain NaphS2 (59, 60). We interpret this as an indication that some of the TRAP transporters of DEH-C11 could allow specific uptake of aromatic substrates from the environment and thereby feed these substrates into the aromatic catabolic pathways described above.

The emerging finding of various genes encoding key enzymes of aromatic-molecule-catabolizing pathways among multiple DEH genomes indicates that catabolism of aromatic molecules may be a common metabolic route for carbon and energy acquisition in various DEH organisms and therefore may be ecologically and biogeochemically important. Sources of aromatic compounds in marine sediments include aromatic amino acids, aromatic fermentation products (e.g., benzoate), or hydrocarbon seeps or spills. Additionally, evidence suggests that a large fraction of the dissolved organic matter pool in the oceans is comprised of diverse aromatic molecules (61), which may be derived from sources such as plant lignin (62, 63) or bacterium-derived metabolites (64). These compounds likely become buried in marine sediments when associated with sinking marine particles. Indeed, recent organic geochemical analyses of subsurface marine sediments have shown that relatively high proportions of lignin-derived aromatic compounds, as well as other aromatic molecules formed by diagenetic processes or of unknown sources, are present in sediments of the Namibian margin (65) or tidal flats of Helgoland, North Sea (66). Diverse aromatic substrates may therefore sustain specialized microorganisms such as the DEH in marine sediments and, importantly, could serve as promising substrates for postgenomic experimentation.

### (ii) Alternative carbon and energy sources.

Several genes that were unique to DEH-C11 versus cultivated DEH gave results that indicate that the capacity to cleave and catabolize different heterocyclic aromatic compounds exists in DEH-C11. These include a gene encoding a putative “creatinine amidohydrolase,” which is known to catalyze hydrolytic cleavage of amide bonds in the heterocyclic compound creatinine, as well as additional genes encoding subunits of a hydantoinase family enzyme (cyclic amidohydrolases) that were detected on contigs not included in the high-confidence data set. Heterocyclic organics that are dearomatized by such enzymes can be converted to central intermediates or directly to amino acids and can also be fermented by some organisms. Additionally, we identified seven predicted proteins with high sequence similarity and conserved domains of the amidohydrolase family that are unique to DEH-C11 versus cultured DEH. Although it is difficult to predict their precise functions, they gave indications of an expanded capacity to hydrolyze organic molecules. The potential to utilize acetone or other short-chain ketones, e.g., butanone, was indicated by genes encoding homologs of alpha and gamma subunits of acetone carboxylases. Gamma subunits of acetone carboxylases are considered to differentiate them from related hydantoinase-type enzymes (67), and phylogenetic analysis also showed that the alpha subunit from DEH-C11 affiliates with known acetone carboxylase-harboring bacteria (see Fig. S6 in the supplemental material). These genes have also been previously identified in RBG-1351 (20). Acetone can be a fermentation product and could therefore be made available by fermenting comembers of the microbial community.

### Cellular functions (cell membrane).

No indications related to peptidoglycan synthesis were found in the DEH-C11 genome content, which is in line with previous studies of DEH isolates and their genomes (22, 54, 68, 69). Various genes encoding enzymes required for the synthesis and modification of glycoproteins, which we hypothesize may be conjugated to surface-layer proteins, were present on a single scaffold (scaffold_7), e.g., genes encoding UDP-glucose 4-epimerase, GDP-l-fucose synthase, GDP-mannose 4,6-dehydratase, and various glycosyltransferases with sequence identities similar to those of various surface-layer-containing archaea. In many surface-layer-containing organisms, such surface-attached glycoprotein chains can have a capsulation effect and may therefore provide extra stability to the cell wall. Such information regarding cell walls could give insights into the ability of DEH to survive in deep marine sediments and into why DEH and *Chloroflexi* are apparently resistant to various cell disruption methods (70) and alkaline lysis procedures in single-cell genome-based studies (18).

### Conclusions.

Our metabolic predictions from the genome of this unique single DEH cell expand our knowledge of the metabolic potential of DEH and the phylum *Chloroflexi* and, notably, provide hints to roles for DEH in the marine sedimentary sulfur cycle. Important information is also provided regarding the existence of a novel clade of RdhA that is mostly derived from DEH, which opens new questions regarding the presence, evolution, and function of reductive dehalogenases in DEH. From an ecological point of view, the predicted ability to respire multiple electron acceptors could enable the bacterium to switch between respiratory modes within heterogeneous marine sediments and/or with burial through different redox zones. The data also add to previous findings that suggest that DEH bacteria can oxidize aromatic compounds. The emerging view from this and other environmental DEH genomic data, as well as 16S rRNA gene sequencing data, suggest varied lifestyles for DEH in marine sediments. Nevertheless, extensive future efforts in genome sequencing will be required in order to understand conserved versus accessory gene information and therefore common versus varied metabolic capabilities among the diverse and yet enigmatic DEH bacteria that exist in the subsurface. Further determining the exact role of DEH in sulfur cycling will also be important, due to the biogeochemical significance of sulfur cycling in marine sediments and the abundance of DEH that are related to the cell analyzed in this study. Finally, this report provides useful information for designing postgenomic experiments to validate predicted metabolic features, e.g., targeted cultivation, experimental transcriptomics and/or stable isotope probing, or combinations thereof.

## MATERIALS AND METHODS

### Sample collection, single-cell sorting, and genome amplification.

Sediment samples were collected from sediments of Aarhus Bay, Denmark (56°9′35.889′′N, 10°28′7.893′′E), from 10 cmbsf in March 2011 (71). Cells were separated from sediment particles by sonication and subsequent density gradient centrifugation in a Nycodenz medium as previously described (71). Methods for cell sorting of fluorescently stained cells, cell lysis, MDA, and screening of single amplified genomes by PCR have been previously described (71). A total of 71 good-quality 16S rRNA gene sequences were recovered from 630 sorted “single cells,” and one of the *Chloroflexi*-related cells was selected for further analysis. The full systematic name of the studied single amplified genome (SAG) is “*Dehalococcoidia* bacterium SCGC AB-540-C11” (abbreviated to “DEH-C11”). The sample processing described here was performed during the same sequence sample processing performed at the Bigelow Laboratory Single Cell Genomics Center (SCGC, https://scgc.bigelow.org/) as previously reported (19, 71). A detailed description of these steps is provided in Text S1 in the supplemental material.

### DNA sequencing and assembly.

Sequencing of MDA-derived DNA was performed using Illumina technology in paired-end mode and 300-bp libraries that were physically “normalized” by a duplex-specific nuclease (Evrogen) treatment in order to reduce the number of DNA fragments from regions of the genome that were amplified more than others by the MDA reaction. Briefly, for 6 µl of DNA sample, 2 µl of 4× hybridization buffer (50 mM HEPES [pH 7.5], 500 mM NaCl) was added. This was incubated at 98°C for 3 min and then at 68°C for 5 h. Soon after, 10 µl of 2× DSN Master buffer (Evrogen), prewarmed at 68°C, was added to the sample and incubated at 68°C for 10 min. After that, 2 µl of duplex-specific nuclease in DSN:Storage buffer mix (1:1), prewarmed at 68°C, was added and the sample was incubated at 68°C for 20 min. Finally, 20 µl of Stop buffer was added to each sample and subjected to vortex mixing. DNA was stored at −20°C. The DNA (20 µl) was then reamplified using Phusion High-Fidelity DNA polymerase (Finnzymes) and the Illumina sequencing adapters as primers with cycling conditions as follows: 98°C for 30 s, followed by 12 cycles of 98°C for 10 s, 65°C for 30 s, and 72°C for 30 s, which was then followed by a final incubation at 72°C for 2 min. PCR products were gel purified and analyzed for size distribution using a Bioanalyzer DNA 1000 chip (Agilent). The DNA was sequenced using an Illumina HiSeq 2000 Sequencer, resulting in 40.8 million reads using 2 × 150-bp mode. Raw reads were trimmed to 101 bp prior to assembly.

Assemblies were performed with SPAdes version 2.3.0 (72) and IDBA-UD version 0.17 (73) using default parameters. Both assemblers were designed to specifically handle sequence datasets from single-cell genomes, i.e., datasets with large differences in genome coverage due to nonuniform amplification of DNA during MDA reactions. Assembly outcomes were compared using QUAST (74). All contigs from each assembly were compared to each other (contigs of >1,000 bp) using BLASTN (75), in order to identify contigs unique to either assembly. SPAdes produced the largest assembly and was used for the main assembly, and the few contigs that were unique to the IDBA assembly were added manually to the SPAdes assembly. All contigs were manually inspected for duplicate or chimeric “mirror-like” formations (produced by SPAdes), and those with such formations were removed.

### Genome annotations.

Initial automatic gene annotations were performed using the RAST server (76) and the MicroScope annotation pipeline (http://www.genoscope.cns.fr/agc/microscope/) (77). Then, all predicted protein sequences were extracted and compared to protein sequences from the previously annotated DEH strains *D. mccartyi* strain CBDB1, *D. mccartyi* strain 195, and *D. lykanthroporepellens* BL-DC-9, separately, using BLASTP and an E value threshold of 10^−10^. All annotations that provided positive hits and revealed the same annotation as the previously annotated reference protein were kept, while discrepancies were manually inspected and edited. All protein sequences were also compared to the NCBI nonredundant (NCBI-nr) database by BLASTP using an E value threshold of 10^−5^ in order to gain hints with respect to the functions of genes not given a function by RAST.

The total genome size was estimated based on numbers of conserved single-copy genes (CSCG) and tRNA genes present in the assembled DEH-C11 data compared to cultivated DEH (19, 78), as well as automatically by CheckM (79).

### Quality control of contigs.

Initial examination of automated annotations identified several small contigs with 16S rRNA genes derived from *Staphylococcus aureus* and *Escherichia coli*, which suggested a contamination of MDA or sequencing reagents since these bacteria are not typically associated with marine sediments. No evidence for contamination from sediment-dwelling microbes could be identified. Most of the contigs contained genes most similar to genes from known DEH organisms and were therefore assigned to the genome of DEH-C11. All other contigs were discarded. Pentanucleotide analysis of contig sequences was performed using VizBin (80) and default parameters, whereby reference genomes were randomly divided into artificial “contigs” of 3 to 8 kbp for analysis and comparison to contigs from DEH-C11. An additional automated check of the assembled genome contamination and completeness was performed by CheckM (79), which identified several possible duplicated contigs, and these were removed.

### Sample collection for long-range *dsr*-locus PCR.

Samples were retrieved in November 2011 from sediments of Aarhus Bay (the same site from which single cells were obtained) at depths of 10 to 20, 30 to 36, 66 to 72, 102 to 108, 135 to 141, 168 to 174, 201 to 208, 235 to 341, 268 to 274, and 301 to 308 cmbsf and from Baffin Bay sediment site 371 (75°58.24′N, 70°34.86′W) at depths of 280 to 300 cmbsf during cruise ARK XXV/3 2010 (81). Tidal flat sediments of the Wadden Sea (54°11'25.6"N, 8°48'51.0"E) were obtained from shallow sediments of 10 to 20 cmbsf. Cells were separated from sediment particles using sonication and density gradient centrifugation as previously described (71). DNA was isolated from the separated cells using a FastDNA Spin Kit for Soil (MP Biomedicals, Eschwege, Germany) according to the manufacturer’s instructions or using a modified chloroform-based protocol (82).

### Long-range PCR amplification and cloning and sequencing of *dsr* loci.

Two different DNA fragments were amplified with sizes of 2.5 and 3.9 kbp, respectively, both coding for *dsr* loci. Details of primers and PCR conditions are provided in Text S1 in the supplemental material. Amplified fragments were cloned using a pGEMT-Easy Vector system (Promega, Madison, WI) or a pJET 1.2 Vector system (Thermo Fisher Scientific, Waltham, MA, USA) according to the manufacturer’s instructions. Clones containing fragments of the expected size were sequenced, and sequences were compared using BLASTN or BLASTX with the NCBI-nr nucleotide and protein databases supplemented with nucleotide and amino acid sequences, respectively, of open reading frames of DEH*-*C11. For selected clones, the full 2.5-kbp or 3.9-kbp insertions were sequenced using internal primers and were assembled with the Sequencher program (Gene Codes Corporation, Ann Arbor, MI, USA). Sequences of the insertions were automatically annotated using RAST 2.0 (83). Alignments were performed using Muscle (84) as implemented in Mega 6.0 (85).

### Phylogenetic analyses.

Phylogenetic analysis of 16S rRNA genes was performed using a curated DEH 16S rRNA gene sequence data set and alignment as described previously (16). The 16S rRNA gene sequence of DEH-C11 was added to the preexisting alignment using MAFFT (86). The alignment was checked and manually curated, and the maximum likelihood algorithm within Mega 6.0 was used for calculating the phylogenetic tree.

For phylogenetic analysis of DsrAB, all unique *dsrAB* sequences obtained in this study were translated and aligned to the curated DsrAB reference alignment previously described (34) within ARB (87). Aligned sequences were then exported while excluding insertion/deletion columns via the SRP_aa filter included with the DsrAB database (34). Phylogenetic placement of sequences into the previously generated DsrAB reference tree “DsrAB_consensus” (34) was performed using the evolutionary placement algorithm (EPA) function of RAxML (88).

For phylogenetic analysis of RdhA, a previously described reference RdhA data set (52) was used as a basis data set. This basis data set was supplemented with RdhA sequences of the best BLASTP hits to the DEH-C11 RdhA from the NCBI-nr database and, additionally, RdhA sequences previously amplified from marine sediments (89). Sequences were aligned with Muscle within Mega 6.0, trimmed at both ends to equal lengths (which included trimming of hypervariable twin-arginine transport [TAT] leader peptide sequence columns), and subjected to the maximum likelihood algorithm using Mega 6.0.

All trees were curated using the Interactive Tree of Life v2 Web-based tool (90).

### Metagenome searching and analysis.

We searched metagenomes publically available from the IMG/M database (http://img.jgi.doe.gov/m) (91) for potentially DEH-derived *dsrAB* and *rdhA* genes by BLASTP using *dsrAB* of DEH-C11 and the reductive dehalogenase gene of DEH-C11 as queries and an E value threshold of 10^−10^. We also searched the database using the full names or modifications of the names of these genes. Focus was placed on the published White Oak River metagenome (51), which was the largest metagenome from estuarine/marine sediments at the time of analysis (December 2014). Contigs with positive hits that were longer than 10,000 bp were retrieved, autoannotated by RAST, and searched for genes of interest. Selected conserved marker genes that were present on the same contigs were analyzed by BLASTP or BLASTN to identify the best hits. Contigs with conserved marker genes with the best hits for DEH and phylogenetic clustering of the corresponding genes with orthologues from *Chloroflexi* were classified as belonging to DEH.

### Search of raw reads of DEH-C11 for unassembled genes related to sulfate reduction.

A search for evidence of genes encoding sulfate adenylyltransferase (SAT) and adenylylsulfate reductase (APS) in the unassembled reads of DEH-C11 was performed in order to examine whether sequence reads with high sequence similarity to these genes were or were not incorporated into the assembly due to low-coverage sequencing. A search of raw Illumina reads that were pretrimmed to 101 bp was performed by using DIAMOND version 0.7.9 (92), whereby a “seed” database of SAT and APS protein sequences from *Desulfosporosinus* spp. and *Desulfotomaculum* spp. was created and used to identify potential reads belonging to *sat* or *aps* genes.

### Nucleotide sequence accession numbers.

The obtained genomic data are being deposited in the GenBank database under BioProject PRJNA303082. Sequences of *dsrAB* sequences affiliated with the *Chloroflexi*-related clade and obtained by PCR amplification are available in the NCBI GenBank database under accession numbers KU561069 to KU561092.

## SUPPLEMENTAL MATERIAL

Figure S1  Pentanucleotide analysis. Download Figure S1, PDF file, 2 MB

Figure S2  16S rRNA gene versus *dsrAB* similarity plot. Download Figure S2, PDF file, 0.5 MB

Figure S3  CISM phylogenetic tree. Download Figure S3, PDF file, 2.5 MB

Figure S4  *rdhA* gene arrangements. Download Figure S4, PDF file, 2.5 MB

Figure S5  NuoL phylogenetic tree. Download Figure S5, PDF file, 1.4 MB

Figure S6  Acetone carboxylase phylogenetic tree. Download Figure S6, PDF file, 1.3 MB

Table S1  Gene list. Table S1, XLSX file, 0.02 MB

Table S2  Long-range PCR *dsrAB* clone sequences. Table S2, XLSX file, 0.02 MB

Text S1  Supplemental materials and methods. Download Text S1, DOCX file, 0.03 MB
