# Supplementary material for: Single-Cell Genome and Group-Specific dsrAB Sequencing Implicate Marine Members of the Class Dehalococcoidia (Phylum Chloroflexi) in Sulfur Cycling
Source: mBio. 2016 May 3;7(3):e00266-16. doi: 10.1128/mBio.00266-16 (PMC4959651; doi:10.1128/mBio.00266-16)
Supplement: Figure S1 — Pentanucleotide analysis. Download [file mbo002162803sf1.pdf]

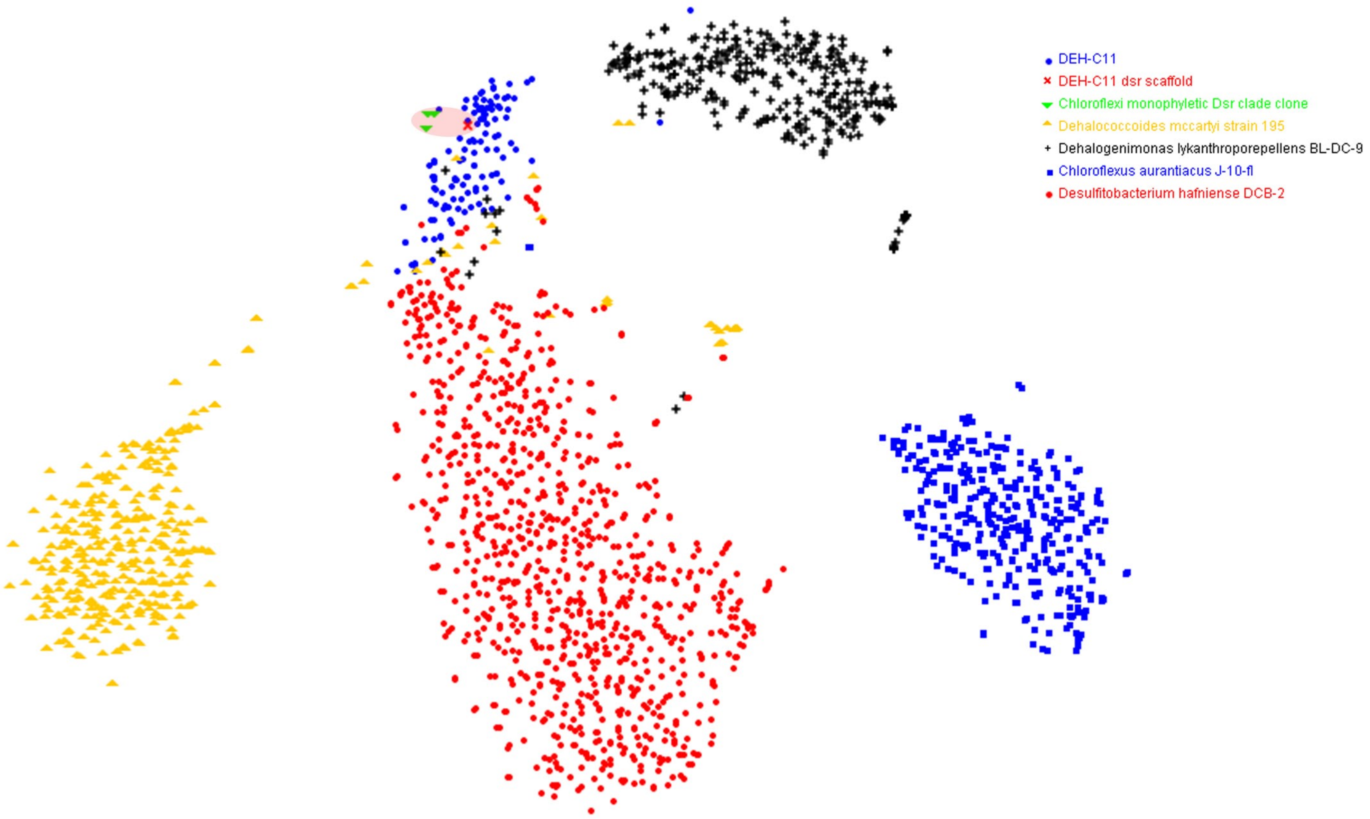

Supplementary Figure 1. Scatter plot of pentanucleotide analysis of contigs performed by VizBin (Laczny et al., 2015) showing DEH-C11 genomic content (blue circles) clusters together. The red shaded elipse highlights the area in which the DEH-C11 *dsr*-harbouring contig (red cross) and other *dsr*-harbouring cloned fragments (green triangles) containing *dsrAB* sequences affiliated with the monophyletic *Chloroflexi* lineage. Each dot represents a contig or scaffold from DEH-C11, or 'artificial' contigs derived from reference genomes after dividing the genome assemblies into 3-8 kbp sequences.
