# Supplementary material for: Single-Cell Genome and Group-Specific dsrAB Sequencing Implicate Marine Members of the Class Dehalococcoidia (Phylum Chloroflexi) in Sulfur Cycling
Source: mBio. 2016 May 3;7(3):e00266-16. doi: 10.1128/mBio.00266-16 (PMC4959651; doi:10.1128/mBio.00266-16)
Supplement: Figure S2 — 16S rRNA gene versus dsrAB similarity plot. Download [file mbo002162803sf2.pdf]

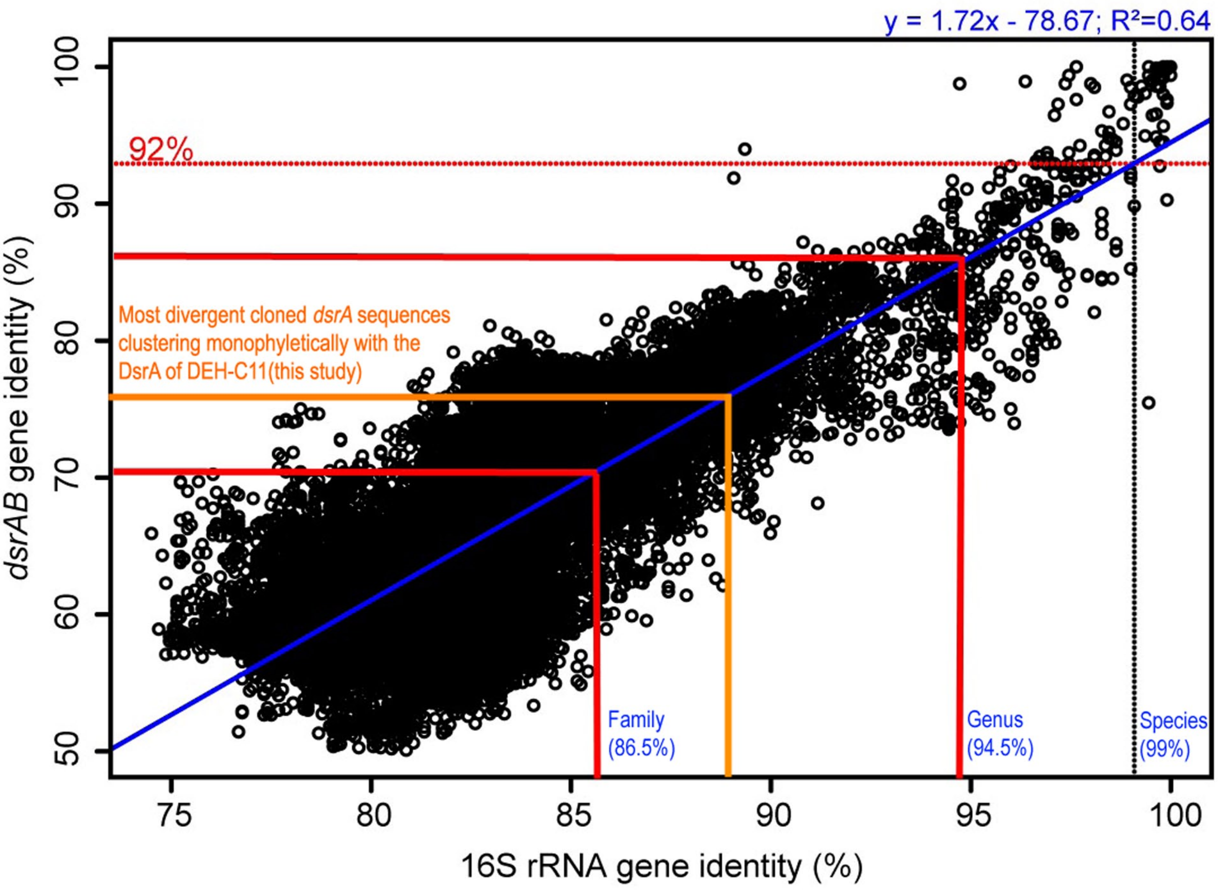

Supplementary Figure 2. Plot showing corresponding nucleotide identities of pairs of dsrAB and 16S rRNA gene sequences from bacteria harbouring non-laterally acquired *dsrAB*. Nucleotide sequence identity cut-off thresholds previously defined to delineate taxonomic boundaries of different species, genera and families for the 16S rRNA gene are indicated (Yarza et al, 2014). The orange line represents the sequence identity of the most divergent *dsrA* sequence obtained in this study that forms a monophyletic clade with the *dsrA* of DEH-C11, to the *dsrA* of DEH-C11. This figure was reproduced and edited from a previous study (Müller et al, 2015), with permission from the authors.
