# Supplementary material for: Single-Cell Genome and Group-Specific dsrAB Sequencing Implicate Marine Members of the Class Dehalococcoidia (Phylum Chloroflexi) in Sulfur Cycling
Source: mBio. 2016 May 3;7(3):e00266-16. doi: 10.1128/mBio.00266-16 (PMC4959651; doi:10.1128/mBio.00266-16)
Supplement: Figure S3 — CISM phylogenetic tree. Download [file mbo002162803sf3.pdf]

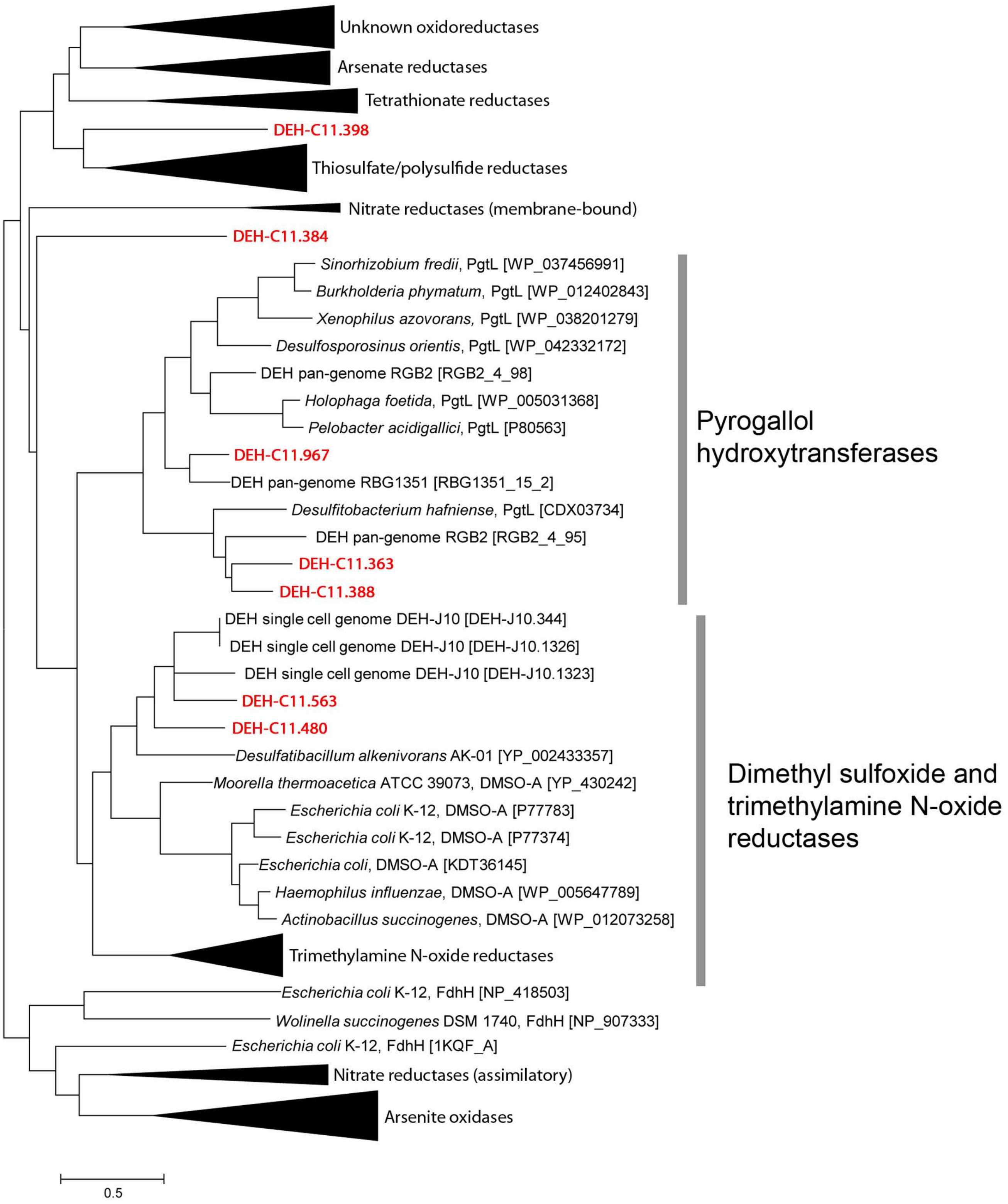

Supplementary Figure 3. Phylogenetic tree based on molybdopterin-containing oxidoreductase 'alpha' subunit protein sequences. Sequences used in the analysis are mostly derived from a previous study (Duval et al, 2008). The tree is based on the Maximum-Likelihood algorithm. Sequences from DEH-C11 are highlighted in red. Major clades containing sequences annotated as having the same function are presented as collapsed branches. Numbers presented in parenthesis represent GenBank accession numbers. The scale bar represent 5% sequence divergence.
