# Supplementary material for: Single-Cell Genome and Group-Specific dsrAB Sequencing Implicate Marine Members of the Class Dehalococcoidia (Phylum Chloroflexi) in Sulfur Cycling
Source: mBio. 2016 May 3;7(3):e00266-16. doi: 10.1128/mBio.00266-16 (PMC4959651; doi:10.1128/mBio.00266-16)
Supplement: Figure S4 — rdhA gene arrangements. Download [file mbo002162803sf4.pdf]

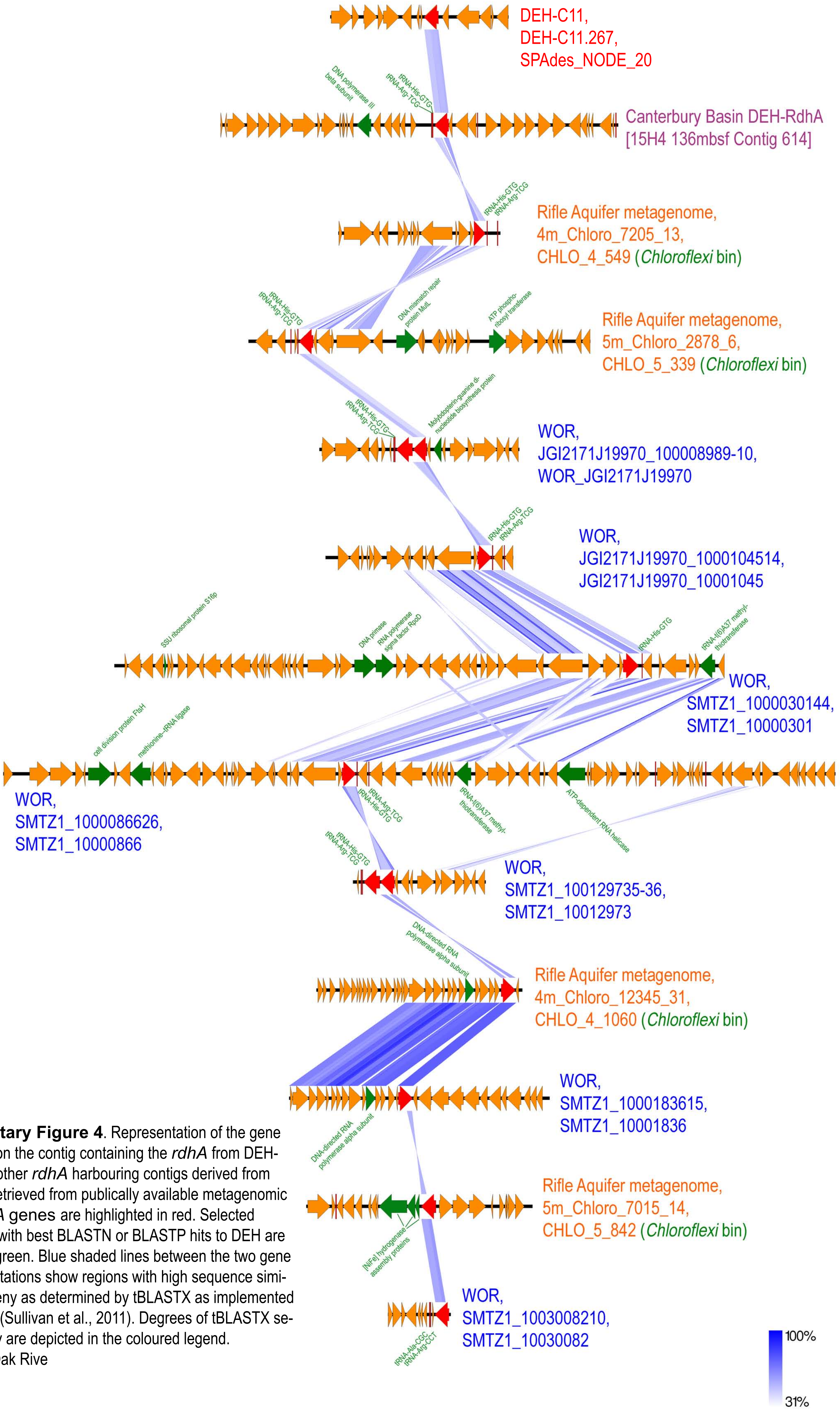

Supplementary Figure 4. Representation of the gene order present on the contig containing the *rdhA* from DEH-C11 and from other *rdhA* harbouring contigs derived from DEH that we retrieved from publically available metagenomic datasets. *rdhA* genes are highlighted in red. Selected marker genes with best BLASTN or BLASTP hits to DEH are highlighted in green. Blue shaded lines between the two gene order representations show regions with high sequence similarity and synteny as determined by tBLASTX as implemented using EasyFig (Sullivan et al., 2011). Degrees of tBLASTX sequence identity are depicted in the coloured legend. WOR=White Oak Rive
