# Supplementary material for: Single-Cell Genome and Group-Specific dsrAB Sequencing Implicate Marine Members of the Class Dehalococcoidia (Phylum Chloroflexi) in Sulfur Cycling
Source: mBio. 2016 May 3;7(3):e00266-16. doi: 10.1128/mBio.00266-16 (PMC4959651; doi:10.1128/mBio.00266-16)
Supplement: Figure S5 — NuoL phylogenetic tree. Download [file mbo002162803sf5.pdf]

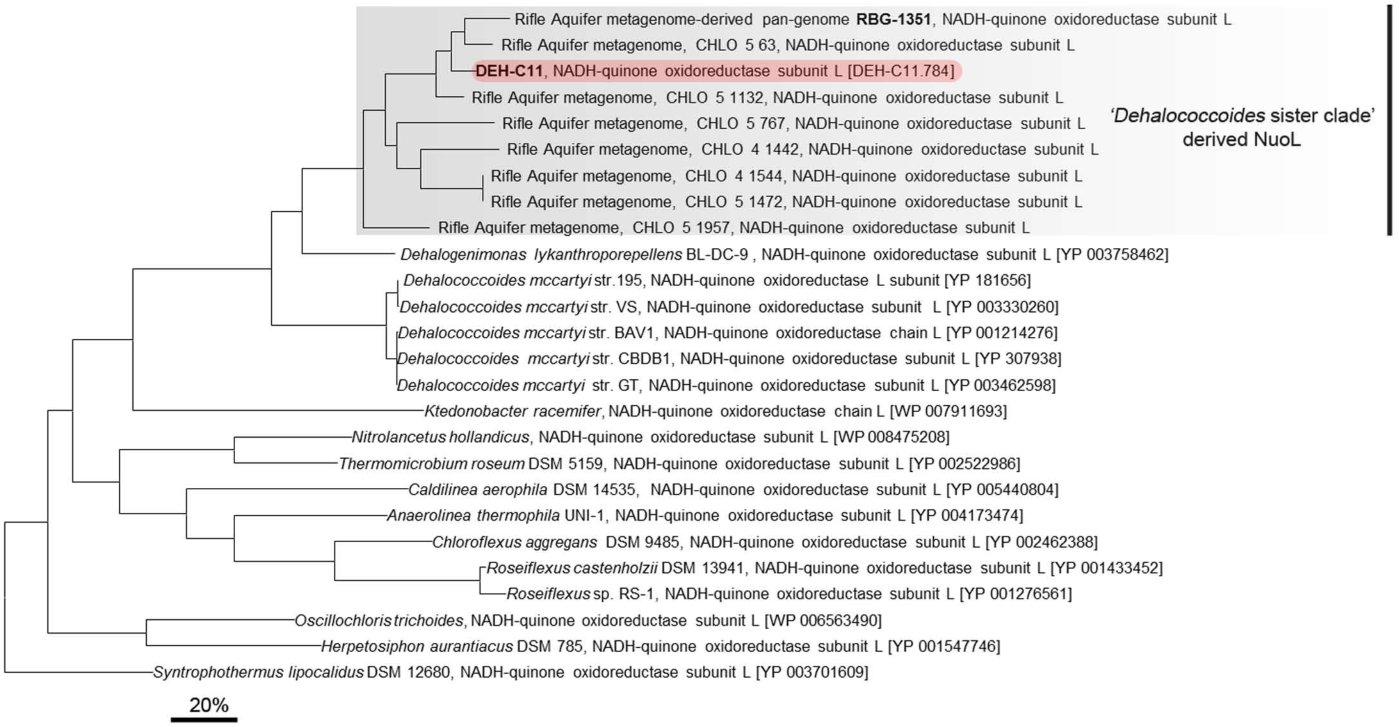

Supplementary Figure 5. Phylogenetic tree based on NADH-ubiquinone oxidoreductase subunit L (NuoL) protein sequences. Sequences used in the analysis are derived from best BLASTP hits to the NuoL of DEH-C11, and also undescribed NuoL sequences from the genome bin of RBG-1351 and additional *Chloroflexi* binned scaffolds from the Rifle Aquifer sediment metagenome (Hug et al, 2013). The tree is based on the Maximum-Likelihood algorithm. The NuoL sequence from DEH-C11 is highlighted in red. Numbers presented in parenthesis represent GenBank accession numbers. The scale bar represent 20% sequence divergence.
