# Supplementary material for: Single-Cell Genome and Group-Specific dsrAB Sequencing Implicate Marine Members of the Class Dehalococcoidia (Phylum Chloroflexi) in Sulfur Cycling
Source: mBio. 2016 May 3;7(3):e00266-16. doi: 10.1128/mBio.00266-16 (PMC4959651; doi:10.1128/mBio.00266-16)
Supplement: Figure S6 — Acetone carboxylase phylogenetic tree. Download [file mbo002162803sf6.pdf]

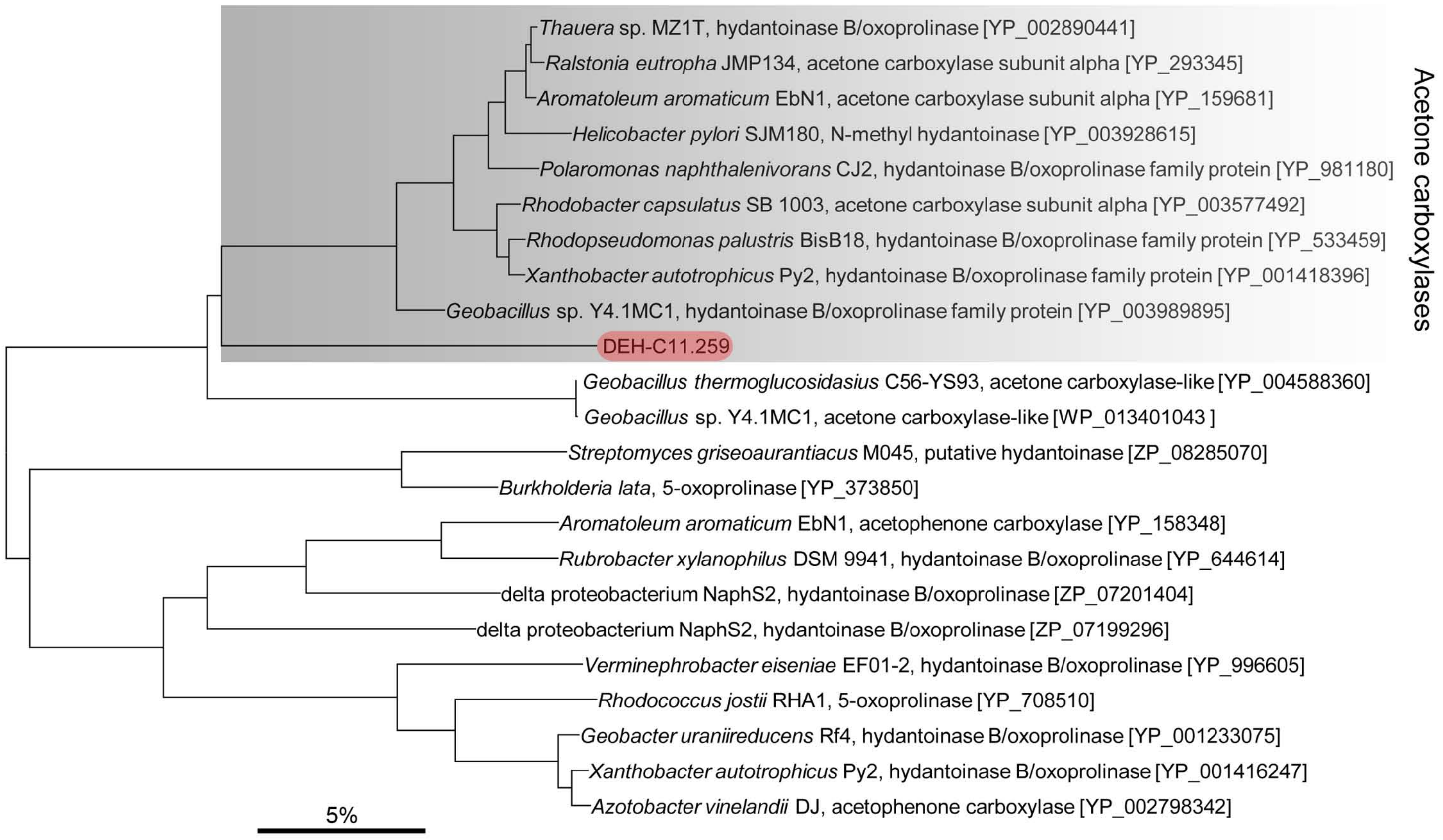

Supplementary Figure 6. Phylogenetic tree based on ATP-dependent hydantoinase, acetone- and acetophenone-carboxylases and corresponding subunits of related enzymes. Reference sequences are derived from a previous study (Schühle and Heider 2012). The tree is based on the Maximum-Likelihood algorithm. Numbers presented in parenthesis represent GenBank accession numbers. The scale bar represent 5% sequence divergence.
