# Supplementary material for: Single-Cell Genome and Group-Specific dsrAB Sequencing Implicate Marine Members of the Class Dehalococcoidia (Phylum Chloroflexi) in Sulfur Cycling
Source: mBio. 2016 May 3;7(3):e00266-16. doi: 10.1128/mBio.00266-16 (PMC4959651; doi:10.1128/mBio.00266-16)
Supplement: Text S1 — Supplemental materials and methods. Download [file mbo002162803s1.docx]

**Supplementary information:**

**Single cell genome and group-specific *dsrAB* sequencing implicate marine members of the class *Dehalococcoidia* (phylum *Chloroflexi*) in sulfur cycling**

Kenneth Wasmund^1,2*^, Myriel Cooper^1*^, Lars Schreiber^3^, Karen G. Lloyd^3,7^, Brett J. Baker^4^, Dorthe G. Petersen^3^, Bo Barker Jørgensen^3^, Ramunas Stepanauskas^5^, Richard Reinhardt^6^, Andreas Schramm^3^, Alexander Loy^2^ and Lorenz Adrian^1^

^1^Helmholtz Centre for Environmental Research – UFZ, Leipzig, Germany.

^2^Division of Microbial Ecology, Department of Microbiology and Ecosystem Science, Research Network Chemistry meets Microbiology, University of Vienna, Vienna, Austria.

^3^Center for Geomicrobiology, Department of Bioscience, Aarhus University, Denmark.

^4^Department of Marine Science, University of Texas-Austin, Marine Science Institute, Texas, USA.

^5^Bigelow Laboratory for Ocean Sciences, East Boothbay, Maine, USA.

^6^Max Planck Genome Centre Cologne, Cologne, Germany.

^7^Current address: Department of Microbiology, University of Tennessee, Knoxville, Tennessee, USA.

* contributed equally.

Corresponding Author: Kenneth Wasmund

Email: kwasmund@gmail.com

# SUPPLEMENTARY MATERIALS AND METHODS

### Sampling, single cell sorting, whole-genome amplification and PCR screening

All methods for sampling of sediments, extraction of cells from sediments and cell separation from sediment particles were conducted as described previously ([Lloyd et al., 2013](#_ENREF_2)), and during the same sampling voyage and processing sequence. A gravity corer was used to collect marine sediment from an area of Aarhus Bay (56°9’35.889 N, 10°28’7.893 E), where the water depth of the sampling site was 16.3 m and an *in situ* temperature of 2.5°C at the sea floor. Cell were extracted and subsequently sorted from a sediment depth of 10 cmbsf. The cell sorting procedure was performed as follows: cells were diluted 1:5 in 1 x PBS to form a slurry. The slurry was sonicated on ice for 2 x 20 s whereby the sonicating probe was placed in the ice-slurry outside of the tube. The slurry was diluted further 1:8 with 1 x PBS, vortexed briefly and larger sediment particles were allowed to settle for 10 min. The collected supernatant was then subject to density gradient centrifugation where 0.75 ml of a 60% Nycodenz solution (w/v) was added below the cell suspension with a needle and syringe, and this was centrifuged at 10500 x g for 60 min at 4°C. The upper phase was collected and 1 x TE and 5% w/v glycerol (final concentration) were added. The collected cells were stored at -80°C and shipped on dry ice to the SCGC in April 2011. Cells were were diluted 1000 x at the SCGC in DNA-free Sargasso Sea water and were then filtered via a 40 µm mesh-size cell strainer (BD). Cells were stained for up to 120 min with SYTO-9 DNA stain (5 µM; Invitrogen) and sorted by a MoFlo™ (Beckman Coulter) flow cytometer using a 488 nm argon laser for excitation, a 70 µm nozzle orifice and a CyClone™ robotic arm for droplet deposition. Cell sorting was based on fluorescence from nucleic acids and side-scatter, and by the ‘purify 0.5 drop’ mode. Gates with high fluorescence yields were sorted to minimize the sorting of autofluorescent sediment particles. Cells were deposited into 384-well plates with 600 nL 1 x TE buffer per well and stored at -80°C until lysis. From the 384 wells, 315 were used for single cells, 66 were used as negative controls (no droplets) and 3 received 10 cells each (positive controls).

Lysis was performed via an initial freeze-thawing treatment (five cycles), as well as further lysis and DNA denaturation treatment by cold alkaline KOH solution according to ([Raghunathan et al., 2005](#_ENREF_3)). DNA was amplified using multiple displacement amplification (MDA) in 10 µL final volume with Repliphi polymerase (Epicentre). MDA reactions were incubated at 30°C for 12-16 h, followed by inactivation at 65°C for 15 min. MDA reactions were monitored by measuring the SYTO-9 fluorescence using a FLUOstar Omega microplate fluorescence reader (BMG Labtech).

Decontamination of workspaces at the SCGC was performed as previously described ([Stepanauskas and Sieracki, 2007](#_ENREF_4)) and included bleaching of sheath lines and futher flushing with DNA-free deionized water. Potential DNA contaminants of MDA reagents were removed by UV treatment ([Woyke et al., 2011](#_ENREF_5)). All cell sorting and MDA reactions were performed in a HEPA-filtered environment.

MDA-derived DNA was screened by PCR with the primers 27F (5’-AGRGTTYGATYMTGGCTCAG-3’) and 907R (5’-CCGTCAATTCMTTTRAGTTT-3’), which target most bacteria ([Lane, 1991](#_ENREF_1)). The primers 27F and 907R were extended by adding the sequencing primers M13F (5’-GTAAAACGACGGCCAGT-3´) and M13R
(5´-CAGGAAACAGCTATGACC-3´), and these were used for priming sequencing reactions ([Lloyd et al., 2013](#_ENREF_2)).

### Long-range PCR amplification of dsr-loci

All PCRs were performed with a Eppendorf Mastercycler® Personal (Eppendorf, Hamburg, Germany) and the Phusion®High-Fidelity DNA polymerase master mix with HF buffer (New England Biolabs, Ipswich, Massachusetts, USA). The final primer concentration in the PCR reaction mix was 400 nM for each. Primers used for long-range PCR fragments of the 3.9 kbp fragments (*dsrABDNC*) contained a phosphorothioate 3’-end to protect from Phusion DNA polymerase exonuclease activity during amplification (Eurofins MWG Operon, Ebersberg, Germany). One µl of DNA sample with a concentration of ~10 ng/µl was added to a PCR reaction with a total volume of 25 µl. The 2.5 kbp fragment coding for *dsrAB-luxR* was amplified using primers C11_luxr1_out1 (5’-CTCTAGAACTTTTCATCCG-3’) and Dsr4R-C11 (5’-GTAAAGCAATTGGCACA-3’), based on sequences of DEH-C11, with the following conditions: 98°C for 15 sec and 35 cycles of 98°C for 15 sec, 54°C for 20 sec and 72°C for 45 sec and a final step of 72° for 5 min. The 3.9 kbp fragment coding for *dsrABDNC* from was amplified with primers dsr1F-DHC-2-pt3 (5’-GATTACACACTGGAATCAyg-3’) and SAG_D_GAMMAF_C (5’-AAGCCATCCTCATCAASyt-3’), or with primers dsr1F-DHC-2-pt2 (5’-GATTACACACTGGAARCAyg-3’) and SAG_D_GAMMAF_B (5’-AAGCCATCCTCATCMASyt-3’). For all primer combinations amplifying the 3.9 kbp *dsrABDNC* fragment the following PCR conditions were applied: 98°C for 30 sec and 30 cycles of 98°C for 10 sec, 58°C for 20 sec and 72°C for 4 min followed by 10 cycles of 98°C for 10 sec, 58°C for 20 sec and 72°C for 5 min, and a final step of 72°C for 10 min. PCR fragments were analysed by electrophoresis in 1% (w/v) agarose gels in 0.5 x TAE buffer (pH 8.3). Up to 10 replicate amplified DNA fragments obtained from samples were combined and gel purified using the Wizard® SV Gel and PCR Clean-Up System kit (Promega, Madison, WI, USA). Clone libraries were constructed using blunt-end PCR products tailed with dATPs, the pGEM®-T-easy vector system (Promega, Madison, WI, USA) and chemically competent NEB C2987 *E.coli* cells (New England Biolabs) according to the manufacturer’s instructions. White clones were picked suspended in PCR grade water and screened for the correct insert size by PCR with M13 vector primers. Plasmids were purified using the NucleoSpin® Plasmid Kit (Macherey-Nagel, Düren, Germany). The full-length sequence of several selected clones was sequenced by GATC European Custom Sequencing Centre (Köln, Germany) using M13 primers and custom internal sequencing primers.

# REFERENCES

Lane, D.J. (1991) 16S/23S rRNA sequencing. In *Nucleic Acid Techniques in Bacterial Systematics*. Stackebrandt, E., and Goodfellow, M. (eds). Chichester, UK: John Wiley and Sons, pp. 115–175.

Lloyd, K.G., Schreiber, L., Petersen, D.G., Kjeldsen, K.U., Lever, M.A., Stepanauskas, R. et al. (2013) Predominant archaea in marine sediments degrade detrital proteins. *Nature* **496**: 215-218.

Raghunathan, A., Ferguson, H.R., Jr., Bornarth, C.J., Song, W., Driscoll, M., and Lasken, R.S. (2005) Genomic DNA amplification from a single bacterium. *Appl Environ Microbiol* **71**: 3342-3347.

Stepanauskas, R., and Sieracki, M.E. (2007) Matching phylogeny and metabolism in the uncultured marine bacteria, one cell at a time. *Proc Natl Acad Sci USA* **104**: 9052-9057.

Woyke, T., Sczyrba, A., Lee, J., Rinke, C., Tighe, D., Clingenpeel, S. et al. (2011) Decontamination of MDA reagents for single cell whole genome amplification. *PLoS ONE* **6**: e26161.
